# New Insights on Plasmin Long Term Stability and the Mechanism of Its Activity Inhibition Analyzed by Quartz Crystal Microbalance

**DOI:** 10.3390/mi13010055

**Published:** 2021-12-29

**Authors:** Marek Tatarko, Ilia N. Ivanov, Tibor Hianik

**Affiliations:** 1 Faculty of Mathematics, Physics and Informatics, Comenius University, Mlynska dolina, 842 48 Bratislava, Slovakia; tatarko4@uniba.sk; 2 Center for Nanophase Materials Sciences, Oak Ridge National Laboratory, P.O. Box 2008, Oak Ridge, TN 37831-6496, USA; ivanovin@ornl.gov

**Keywords:** plasmin, trypsin, α_2_-antiplasmin, inhibition, research quartz crystal microbalance, food quality, milk

## Abstract

We used the research quartz crystal microbalance (RQCM) to monitor regulatory effects of plasmin and trypsin in the presence of their inhibitor α_2_-antiplasmin. The gold surface of quartz crystals was modified with a β-casein layer that served as a substrate for protease digestion. The addition of plasmin or trypsin as well as their mixtures with α_2_-antiplasmin resulted in an increase of resonant frequency, f, and in a decrease of motional resistance, R_m_, depending on the molar ratio of protease: antiplasmin. At equimolar concentrations of protease and α_2_-antiplasmin (5 nM:5 nM) full inhibition of protease activity took place. Monitoring of plasmin activity on an hourly and daily basis revealed a prominent effect of autolysis and decrease of plasmin activity in freshly activated samples. The degree of inhibition as well as plasmin half-life (t_1/2_ = 2.48 ± 0.28 days) connected with its degradation was determined.

## 1. Introduction

Diversification of suppliers in food production while assuring high quality food standards requires sensitive techniques for monitoring even slight changes in food composition. This is particularly important for dairy products that usually have a short shelf life and are more liable to changes in their composition and taste [1]. To sustain product quality, one must apply suitable techniques to monitor possible ongoing changes in its composition. Main components providing the unique taste of the milk and milk products are α, β and κ- caseins, milk proteins that exist in micellar form [2,3]. Collapse of organized casein micelles leads to milk gelation and release of peptide fragments, which results in milk bitterness [4]. Casein can be cleaved by plasmin. Ultra-Heat Treatment (UHT) is used to minimize plasmin activity in the milk [5]. However, due to the thermostability of plasmin as well as its activators and inhibitors, even residual concentrations of plasmin can trigger undesired changes of milk quality [6,7]. One plasmin deactivation mechanism is the plasmin protease system which can inhibit plasmin activity indirectly or directly through blocking of plasmin activators inhibitor (PAI) I and II or α_2_-antiplasmin, respectively [8,9]. Another plasmin deactivation process involves whey proteins and plasmin substrates [10]. The α_2_-antiplasmin, for example, binds to the plasmin through lysine residues of Kringle domains with the formation of plasmin-antiplasmin complex, which is associated with the regulation of fibrin cleavage. Monroy and Ruiz [11] proposed that the envelope glycoprotein of the dengue virus can participate in hemorrhagic phenomena by activation of plasmin zymogen—the plasminogen, and thus initiating fibrinolysis. This process was prevented by α_2_-antiplasmin.

Due to the rather low concentration of plasmin in milk (1.65–8.53 nM) [12], sensitive methods are required to detect residual (sub nanomolar) plasmin concentrations. It is also very important to detect small changes in plasmin activity caused by inhibitors, activators and other nonspecific factors affecting the proteolysis of caseins [13]. Detection of plasmin was so far focused mostly on the bloodstream environment. In this case, the plasminogen and plasmin assist in the dissolution of blood clots due to cleavage of fibrin. The first method of plasmin determination was based on radio detection using measurements of γ radiation with photon energy of 140 keV produced by technetium (^99m^Tc)-labeled plasmin. Despite the high sensitivity of this method allowing detection of the plasmin below 1.34 μM, the specificity was low [14]. Clotting and the degree of fibrinolysis effected by plasmin inhibition was determined by detection of γ-rays from ^125^I-labeled substrates. The limit of detection (LOD) in this case was 1.7 μM [15]. The fluorometric method was successfully used to detect elevated plasmin activity that correlated with dry eye syndrome [16,17]. The LOD of the above method was approximately 1.81 μM for certain groups of patients [18]. Recent research on plasmin detection by Dacres et al. [12] reports a sensitive biosensor based on bioluminescence resonance energy transfer (BRET) that achieved plasmin detection with an LOD of 0.25 nM for human plasmin and 0.86 nM for the bovine plasmin in milk, respectively. Most recently, surface-enhanced Raman scattering (SERS) has been reported by Yazgan et al. [19] for detection of plasmin activity using peptide substrate with a good sensitivity of 6.42 U/mL and thus the possibility of plasmin detection in milk. Calorimetric tests were also developed for monitoring of increased levels of plasmin due to bovine mastitis with a sensitivity of 1 ng/mL [20]. Another method of plasmin detection using the switchable peroxidase-mimicking activity of gold nanoparticles coated by β-casein achieved detection of plasmin with a LOD of 44 ng/mL and proved the possibility of protease detection in spiked UHT milk [21].

Due to the rather complex functionality of the plasmin system, which involves activators and inhibitors of plasmin activity, it is also desirable to detect plasmin activity in the presence of inhibitors. Plasmin inhibition was detected using changes in absorbance at 405 nm during plasmin cleavage of D-Val-Leu-Lys-p-nitrophenylanilide substrate for dengue fever assay using enzyme-linked immunosorbent assay (ELISA). This assay is based on measurements of the inhibition effect of antibodies for dengue type 4E protein. The LOD for plasmin in this case was about 60 nM [22].

Electrochemical methods of plasmin detection allowed improving the sensitivity and simplicity of analysis. For example, amperometric detection of plasmin uses short peptides (modified at the N-end by ferrocene (Fc) redox label) chemisorbed by cysteine residues at their C-end on the surface of gold electrodes. Addition of plasmin resulted in cleavage of the peptide and removal of the Fc-contained fragment. This was reflected in decrease of the current. The LOD was in this case 0.6 nM [23]. This method was also used for detection of plasmin in a real milk demonstrating an LOD of about 0.56 nM [24].

Surface-sensitive acoustic methods are rather useful for analysis of various processes [25]. They can also be used for detection of protease activity because they do not require any labeling of the substrate [26]. In our previous work, the thickness-shear mode (TSM) method has been used to monitor cleavage by plasmin of the short peptides at the surface of piezocrystal. The peptides were chemisorbed at the gold surface of the piezoelectric transducer (AT-cut quartz crystal with fundamental frequency of 8 MHz). The increase of resonant frequency, f, and decrease of motional resistance, R_m_, were used to measure corresponding changes in the mass or thickness and viscosity contribution of the peptide layer upon cleavage of peptide substrate by plasmin, with a LOD of 0.65 nM [27]. We showed recently that a multiharmonic quartz crystal microbalance (QCM) biosensor together with a machine learning algorithm can be used to detect residual plasmin activity with an LOD of 0.5 nM [28]. The electromagnetic piezoelectric acoustic sensor (EMPAS) can be also used to detect sub nanomolar concentrations of plasmin in solution, with an LOD of about 32 pM [29]. Most recently, we performed comparative analysis of plasmin detection by QCM and ELISA methods with similar LODs of 167.16 ± 39.36 pM and 121.98 ± 18.30 pM, respectively. However, in the case of ELISA, only the whole plasmin concentration, but not its cleavage activity, was determined [30]. In addition to surface-sensitive acoustic methods, we also applied high-resolution ultrasonic spectroscopy for detection of trypsin [31] and plasmin [32], using β-casein as a substrate. Ultrasonic spectroscopy is based on measurement of the changes in the ultrasound velocity and attenuation in the cells, using a volume of approximately 0.7 mL. The cleavage of the casein resulted in the appearance of short peptides, which increases the degree of the well-ordered hydrated shell surrounding the proteins and increases the ultrasound velocity. The ultrasound spectroscopy permits detection of trypsin and plasmin in sub nanomolar concentrations, similarly to the surface acoustic methods.

While there are several plasmin detection techniques as described above, most of them are used for plasmin detection in other body fluids, mainly blood, and are not suitable for the testing of milk. The prime factor is the insufficient LOD for UHT-deactivated plasmin levels and the high expenses for their regular daily use, as a large proportion of milk worldwide comes from small-scale producers, who are dominant in developing countries [33]. Most of these producers do not have facilities for high throughput quality testing [30]. In addition, most of the techniques available for routine tests, such as ELISA, can detect only whole plasmin, but not its activity [30]. The commercially available assays for plasmin activity determination are based on fluorescence or colorimetric detection, which are not suitable for direct measurements in non-transparent liquids such as raw milk [34].

Acoustic methods have several advantages, including reusable sensor substrate, label-free detection, operation in non-transparent liquids, capability to evaluate viscoelastic properties in solution or at surfaces, and possibility of combining measurement with other techniques [35,36,37,38].

Here, we report the results of our study of the effect of plasmin inhibitor (α_2_-antiplasmin) on the cleavage of plasmin and trypsin, and analysis of long-term stability of plasmin (without inhibitors), using the acoustic research quartz crystal microbalance (RQCM) method, based on β-casein layers that serve as a substrate for plasmin cleavage. To the best of our knowledge, this is the first report on the application of acoustic methods to study plasmin inhibition and its long-term stability.

## 2. Materials and Methods

### 2.1. Reagents

All chemicals were of p.a. grade and used without further purification. Experiments were performed in 10 mM phosphate buffered saline (PBS), pH 7.4. To prepare PBS, 10 mM Na_2_HPO_4_, 1.8 mM KH_2_PO_4_, 137 mM NaCl and 2.7 mM KCl (Slavus, Bratislava, Slovakia) were diluted in ultrapure deionized water prepared by PureLab Classic UV (Elga Water Systems, Buckinghamshire, UK). PBS solution was constantly stirred for at least one hour and adjusted to pH 7.4 using pH meter FiveEasy FE20 (Mettler Toledo AG, Greinfensee, Switzerland). Then, the PBS was filtered using 0.22 μm pore size filters (Millipore, Burlington, MA, USA) and divided into 50 mL fractions that were frozen. Each fraction was thawed just before use. 10 mM PBS provides a good medium for dilution of even higher β-casein concentrations.

Plasmin was prepared by mixing of plasminogen (Sigma-Aldrich, Darmstadt, Germany) in 10 mM PBS with the urokinase activator (Merck, Darmstadt, Germany) which was diluted to 10 kU/mL in deionized water. The concentration of plasmin was determined spectroscopically by measuring plasmin activity using spectrozyme-PL (Sekisui Diagnostics, Burlington, MA, USA) in TRIS buffer by means of UV-Vis spectrophotometer UV1700 (Shimadzu, Kyoto, Japan) according to the method described by Kolev et al. [39]. TRIS solution for spectroscopic measurements was prepared using 20 mM TRIZMA (Sigma Aldrich, Darmstadt, Germany) and 150 mM NaCl (Slavus, Bratislava, Slovakia) in deionized water. The solution was adjusted to pH 7.4 and filtered using a 0.22 μm filter (Millipore, Burlington, MA, USA). 50 mL TRIS fractions were frozen until use. In the experiments, we also used trypsin, β-casein, α_2_-antiplamin, 11-mercaptoundecanoic acid (MUA), 1-Ethyl-3-(3-dimethylaminopropyl) carbodiimide (EDC) and N-hydroxysuccinimide (NHS), (Sigma-Aldrich, Darmstadt, Germany).

### 2.2. Preparation of β-Casein Layers and RQCM Measurements

The gold electrode surface of piezoelectric transducers (AT-cut crystals with fundamental frequency 5 MHz, Total Frequency Control Ltd., Storrington, UK) was carefully cleaned prior to preparation of β-casein layers, using basic Piranha (5:1:1 v/v mixtures of H_2_O, 30% H_2_O_2_, 70% HNO_3_) heated to 70 °C. QCM transducers were immersed in this solution for three 20 min cleaning cycles. After each cleaning cycle, the crystal was washed three times with deionized water and immersed back into the vial with new Piranha solution. Finally, the crystals were washed in deionized water and stored in 96.6% ethanol (Slavus, Bratislava, Slovakia). For preparation of β-casein layers, the crystal was first dried in a stream of nitrogen and then immersed in 2 mM MUA solution in ethanol for 16 h. After rinsing with deionized water, the crystal was immersed in a mixture of 20 mM EDC and 50 mM NHS for 20 min. This was necessary for activation of carboxylic groups of MUA. Finally, the crystal was washed by H_2_O and inserted into the flow cell [40]. The β-casein layer was prepared by the flow of the 1 mg/mL of β-casein in 10 mM PBS using GeniePlus syringe pump (Kent Scientific, Torrington, CT, USA) with a flow rate of 50 μL/min. Because the concentration of β-casein was above its critical micelle concentration (CMC) (CMC of β-casein is 0.5 mg/mL) [41], the solution sample contains casein micelles and produced a thick and stable layer on the activated MUA surface.

The measurements of resonant frequency, f, and motional resistance, R_m_, were performed using Research Quartz Crystal Microbalance (RQCM) (Inficon, Syracuse, NY, USA). According to the Sauerbrey equation, the change in the resonant frequency of RQCM is proportional to the adsorbed or removed mass, as described:

Δf = −2f_o_^2^Δm/A(μ_q_ρ_q_)^1/2^ (1)

where f_o_ is the fundamental frequency of the crystal, Δm is the mass change, and A is the area of the working electrode (in our case 0.2 cm^2^); μ_q_ = 2.95 × 10^11^ dyn/cm^2^ and ρ_q_ = 2.65 g/cm^3^ are the shear stiffness and mass density of the quartz crystal, respectively [42]. The resonant frequency reflects the changes in mass or thickness of the β-casein layer. The RQCM measurements provide information on the motional resistance, R_m_, related to the changes in viscosity [43]. Before plasmin application, a long stabilization period was required for removal of unbound β-casein molecules from the crystal surface. For this purpose, the PBS was allowed to flow along the crystal surface for at least 35 min at the rate of 50 μL/min. Once the resonance frequency was stabilized, PBS was exchanged for 2 mL of the protease sample. This part of the measurement was the most critical and the QCM cell was regularly checked for air bubbles or other influencing factors as the pressure of the pump syringe and solution temperature. The protease was delivered through a flow stream to the surface of the QCM transducer with a rate of 50 μL/min for 35 min. Then, the protease solution was exchanged for PBS and flowed for at least 20 min in order to remove cleaved residues of β-casein. Data acquired from RQCM measurements were evaluated using the OriginPro version 7.5 (OriginLab Corporation, Northampton, MA, USA).

### 2.3. The Concept of the Analysis of Protease Inhibition

Plasmin and trypsin are serine proteases that cleave proteins by hydrolyzing the peptide bonds on determined sites. Blocking such sites prevents access of proteases to them. However, more often than blocking of the substrate, it is the binding of an inhibitor to the protease that prevents substrate cleavage. Detection of the protease activity using acoustic methods provides more detailed information about effect of inhibitors when compared to electrochemical or optical methods, by eliminating the effect of the cleaved fragments on the measuring parameters. Therefore, we used the RQCM method to study the effect of α_2_-antiplasmin on the inhibition of plasmin. α_2_-antiplasmin is the main plasmin inhibitor in the process of fibrinolysis. This inhibitor forms a 1:1 stochiometric complex with plasmin, interacting at its active site with Met377–Arg376 and binding to complementary lysine binding sites of Kringle domains with its lysine residues [44]. The QCM method and derived techniques already showed their effectiveness in detecting uninhibited plasmin. It was able to reach high sensitivity with sub nanomolar concentrations using TSM or EMPAS methods, as has been mentioned in the Introduction.

**Figure 1:**
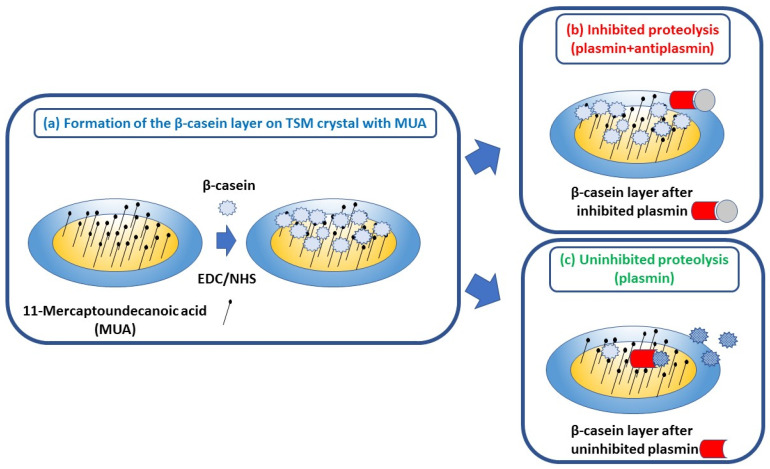
The scheme of the RQCM assay for detecting plasmin inhibition by α_2_-antiplasmin. (**a**) Gold surface of QCM crystal with chemisorbed MUA. Free carboxylic ends are activated by NHS/EDC. Addition of β-casein resulted in its covalent immobilization; (**b**) α_2_-antiplasmin resulted inhibition of β-casein cleavage by plasmin; (**c**) cleavage of β-casein in the presence of plasmin only.

Based on previous experiments [28,29,32], β-casein has been selected as an optimal substrate for detection of plasmin activity. Moreover, β-casein naturally presents in milk and therefore provides more realistic conditions for plasmin detection. While direct β-casein immobilization would be an option also for acoustic biosensors, application of standards in the dairy industry requires a stable layer that could be prepared rather quickly. Therefore, we applied the flow setup that is suitable for QCM crystal with a gold surface covered by MUA layers activated by NHS/EDC chemistry. This is rather simple procedure that enables formation of a stable and oriented protein layer in a shorter time in comparison with procedures based on chemisorption of short peptide layers. The basic principle of RQCM assay for detection of plasmin inhibition by α_2_-antiplasmin is shown in Figure 1.

It is also known that during longer storage any plasmin molecule with an unmodified K4 Kringle domain will undergo autolysis which reduces its activity. Removal of this domain from the plasmin structure causes significant decrease in plasmin autolysis [45]. We used the RQCM method to study the effect of plasmin autolysis by monitoring the cleavage of β-casein in the presence of 5 nM plasmin for 10 days. The plasmin aliquot, activated as described above, was stored at 4 °C and its effect on the β-casein layer was observed for 10 days without inhibitor.

## 3. Results and Discussion

### 3.1. Inhibition of Trypsin and Plasmin by α_2_-Antiplasmin

**Figure 2:**
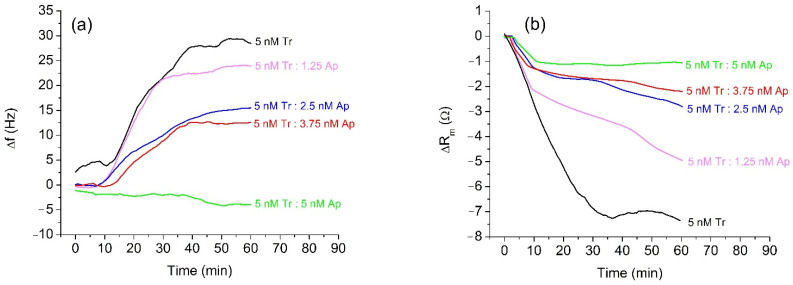
Changes in the resonant frequency, Δf, (**a**) and motional resistance, ΔR_m_, (**b**) of QCM transducer during cleavage of the β-casein layer by 5 nM trypsin (Tr) and at various concentrations of α_2_-antiplasmin (Ap).

In the first series of experiments, we used trypsin as a model protease for the study of the effect of α_2_-antiplasmin inhibition. The advantage of trypsin is that it does not require activation and its concentration is more stable over time, while α_2_-antiplasmin has a similar effect on trypsin and plasmin [46]. Trypsin cleaves peptides on the C-terminal side of Lys and Arg at optimal pH between 7 and 9. Compared to plasmin, it is stable for prolonged storage. The protease concentration in our measurements was around 5 nM, which is within the physiological range of plasmin concentration in milk. Therefore, 5 nM trypsin was incubated for 20 min with α_2_-antiplasmin at various trypsin: antiplasmin molar ratios (4:0, 4:1, 4:2, 4:3, and 4:4). The concentration of inhibitor varied in the range 0–5 nM. Each concentration of α_2_-antiplasmin was used in a separate measurement on a QCM crystal with a freshly prepared β-casein layer. As the α_2_-antiplasmin binds similarly to the other serine proteases, we expected that it will also bind trypsin and cause inhibition as for plasmin. The sensitivity of the RQCM assay was indirectly tested by increasing the α_2_-antiplasmin concentration, which should decrease the amount of unbound protease capable of β-casein cleavage. We assume that unbound protease in the sample with 5 nM α_2_-antiplasmin is below nanomolar level. Figure 2a shows the changes of the resonant frequency, Δf, of QCM transducer at presence of 5 nM trypsin at various concentrations of α_2_-antiplasmin. It is evident that addition of 5 nM trypsin resulted in a rapid increase in resonant frequency, with subsequent stabilization after approximately 35 min. The maximal frequency change was about 29.4 Hz. The frequency increase is due to the removal of β-casein fragments from the surface of QCM transducer. The β-casein layer becomes thinner due to loss of mass. Thus, the increase of the frequency agrees well with the Sauerbrey Equation (1).

The presence of α_2_-antiplasmin resulted in smaller frequency changes compared to those of non-inhibited trypsin. With the increased concentration of α_2_-antiplasmin, changes of resonant frequency, Δf, decreased. At an equimolar mixture of trypsin and α_2_-antiplasmin (5 nM Tr:5 nM Ap), only a small decrease of frequency, f, was observed. This indicates that α_2_-antiplasmin fully inhibited the trypsin activity; the RQCM does not register the presence of the inhibited trypsin because no β-casein is being cleaved. The decrease in frequency is probably due to nonspecific adsorption of the inactivated complexes of trypsin and α_2_-antiplasmin to the surface of β-casein or unblocked MUA. In an independent experiment, we confirmed that application of the pure α_2_-antiplasmin did not cause frequency change. We used the RQCM method to analyze also the changes of motional resistance, ΔR_m_, at various trypsin: α_2_-antiplasmin ratios. The changes of R_m_ values during the cleavage of β-casein by trypsin without and in the presence of α_2_-antiplasmin are shown in Figure 2b. In the presence of 5 nM trypsin, a substantial decrease in the value of R_m_ occurred. This can be related to an increase of molecular slip between the protein layer and surrounding PBS. The reason for this effect may be to the removal of peptide fragments from the β-casein layer, which can decrease the contribution of viscosity. It can be also seen that increased concentrations of α_2_-antiplasmin caused decrease in the motional resistance.

**Figure 3:**
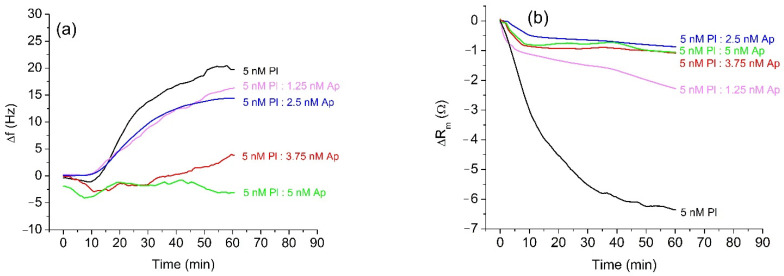
Changes in the resonant frequency, Δf, (**a**) and motional resistance, ΔR_m_, (**b**) of QCM transducer during cleavage of the β-casein layer by plasmin (Pl) at 5 nM of protease and at various concentrations of α_2_-antiplasmin (Ap).

The results of measurements of the β-casein cleavage by plasmin were similar to those with trypsin. Before the measurement, plasmin was freshly activated, and its concentration was determined by a standard spectroscopic method using Spectrozyme-PL as a substrate. A diluted sample of 5 nM plasmin was incubated for 20 min with α_2_-antiplasmin at concentrations in the range 0–5 nM and was applied to the β-casein layer. The changes of the resonant frequency presented on Figure 3a are similar to those for trypsin. The full inhibition of the cleavage of β-casein occurs at an equimolar ratio of plasmin (Pl) and α_2_-antiplasmin (Ap) (5 nM Pl:5 nM Ap), which agrees well with the 1:1 molar ratio of plasmin to α_2_-antiplasmin reported in the literature [9]. The changes of motional resistance were similar to those with trypsin (Figure 3b). However, non-inhibited plasmin caused disproportionately higher changes in R_m_ value in comparison with samples containing α_2_-antiplasmin. The quantitative analysis of inhibition for different concentrations of α_2_-antiplasmin in trypsin and plasmin samples is presented in Table 1. The measured frequency shifts were also used to estimate the changes of the mass of β-casein at the crystal surface using the Sauerbrey Equation (1). With increased concentration of α_2_-antiplasmin, the changes in mass density decrease. This corresponds to the increase of the degree of inhibition of the protease’s activity. One may mention that the Sauerbrey equation is strongly valid only for dry layers in a vacuum. Therefore, the values for Δm/A represent only rough estimates. Despite this, a clear tendency is observed.

**Figure 4:**
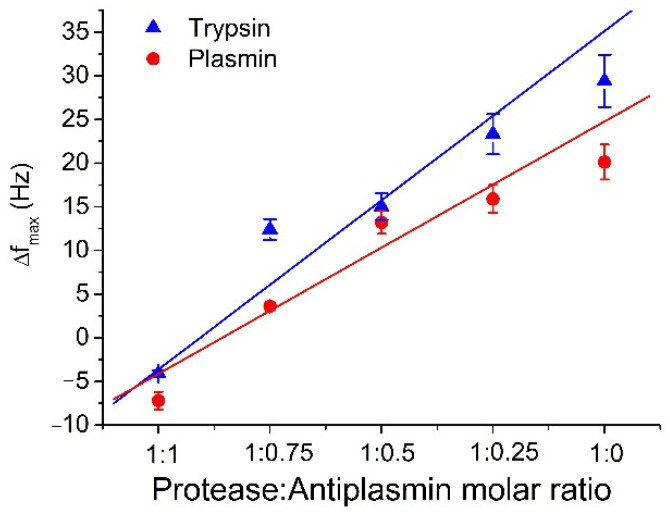
The comparison of the maximal changes of the resonant frequency, Δf_max_, for different molar ratio of protease: α_2_-antiplasmin for trypsin and plasmin (see the legend). The results represent the mean ± SD obtained from 3 independent experiments.

The comparison of the degree of inhibition of trypsin and plasmin by antiplasmin is presented in Figure 4. The changes in frequency due to cleavage of β-casein by plasmin are lower than those for trypsin. This is because the sites of cleavage on the β-casein are more specific for plasmin than for trypsin [47]. In addition, because the number of cleavage sites for trypsin is higher than for plasmin, even a low concentration of active trypsin is able to efficiently cleave β-casein. In the experiments, we used freshly prepared and aged plasmin. Slight variations were observed in measured data when the same plasmin sample was reused for consecutive measurements. This effect is due to the changes of plasmin activity during its longer storage. When older plasmin was used, we observed a decrease in the sensor response. The study of this effect is crucial for functionality of the biosensor, so that we can predict and expect lower values of frequency change when old sample is used.

### 3.2. Monitoring of the Plasmin Autolysis

**Figure 5:**
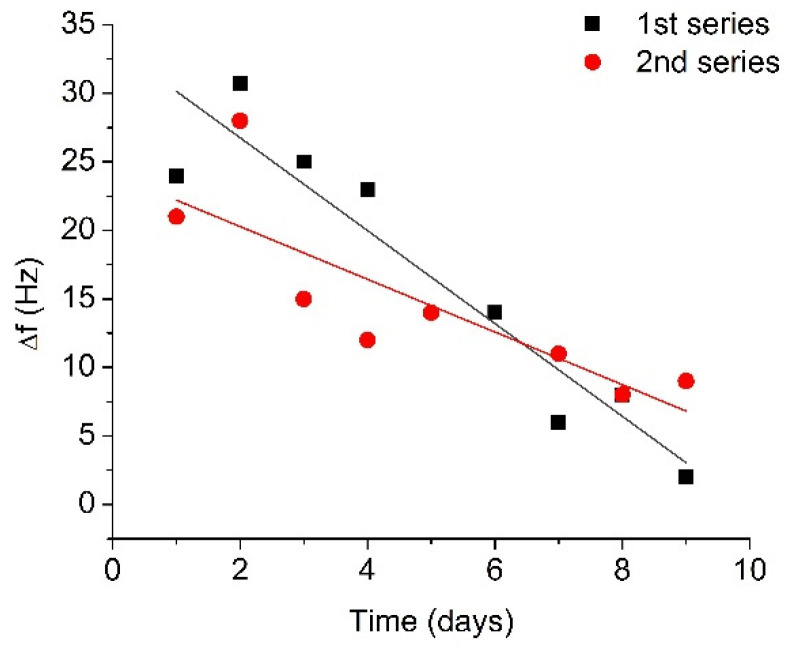
Changes in the resonant frequency caused by addition of activated plasmin (concentration 5 nM) at the surface of the β-casein layer, measured for several days after the initial activation and storage at 4 °C.

The results for two 10-day series of RQCM measurements are presented in Figure 5 as a plot of the changes of resonant frequency vs. time. Figure 5 shows that the average changes of resonant frequency corresponding to the cleavage of β-casein for the fresh plasmin (first day of measurements) were 22.6 Hz. On the second day after plasmin activation, higher frequency changes were observed compared to the first day (29.4 Hz), in both series. In subsequent measurements, a recovery of the frequency shift took place, and after 6 days the frequency shift had recovered by approximately 60% in comparison with the changes on the first day. After 9 days, the resonance frequency changes were 5.5 Hz, which is less than a quarter of the original frequency shift. In fact, the decrease of the degree of β-casein cleavage was due to the decrease of plasmin activity associated with autolysis. The obtained results can be analyzed in the framework of the plasmin shelf time. The reaction rate of plasmin decay can follow the integrated form of the general reaction scheme [Pl] = [Pl]_0_e^-kt^ (where [Pl] is plasmin concentration and [Pl]_0_ is initial plasmin concentration). Using this equation and fitting exponential decay of frequency response using OriginLab software, the coefficient k has been estimated as k = 0.278 ± 0.036 day^−1^. Thus, the plasmin half-life from fitted curve would be t_1/2_ = 2.48 ± 0.28 days (See Supplementary Material for determination of k and t_1/2_ values). This is the time needed for plasmin to decrease to half its original concentration. If the time of the initial increase of the plasmin concentration is omitted from analysis process, one will be able to obtain correlation with the previously reported plasmin shelf half time of 2–2.5 days in blood [48].

The most interesting part of the frequency dependence in Figure 5 is the initial increase in the resonant frequency, which can be seen in both series of measurements. One can speculate that due to a relatively high concentration of plasmin and possible presence of non-activated plasminogen, the residual plasminogen can be transformed into active plasmin. This effect may cause the increase in the resonance frequency during the first two days due to an increased concentration of plasmin in the solution. Thus, the RQCM method is sensitive for detection of the urokinase-type activator which cleaves residual plasminogen in solution and leads to an increase in plasmin concentration. The protease autolysis was studied earlier in the work of Thomas et al. [49] by SDS-PAGE and capillary liquid chromatography to improve the detection of the prohibited substances in sport. They were able to detect approximately 200 nM trypsin and other proteases, while RQCM methods allowing detection of sub nanomolar protease concentrations.

**Figure 6:**
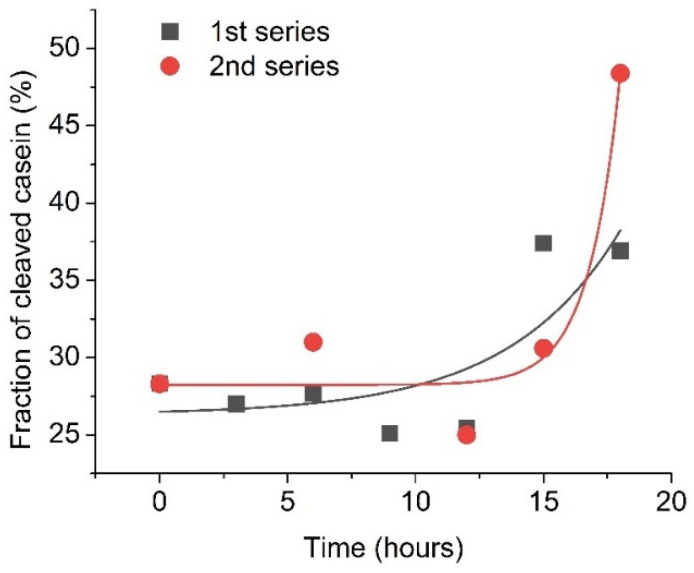
Fraction of the cleaved β-casein as a result of addition of activated plasmin on the surface of β-casein, measured during several hours after initial plasmin activation. The initial plasmin concentration was 5 nM. Solid lines are exponential fit of data.

Furthermore, plasmin was also monitored approximately each 2–3 h in two 20-hour series of RQCM measurements to confirm early changes in its concentration. In these experiments, 5 nM of plasmin from a single activated batch was applied at the surface of a QCM transducer containing a β-casein layer and the changes of resonant frequency were monitored immediately after plasmin activation and also 3, 6, 9, 12, 15 and 18 h after its activation. The results are presented in Figure 6. To improve the precision of plasmin detection, the fraction of cleaved β-casein has been determined by calculating the ratio of resonance frequency changes for β-casein layer cleavage by plasmin (Δf_Plasmin_) to the resonance frequency changes for formation of the β-casein layer (Δf_casein_). The fraction of cleavage of casein is (Δf_Plasmin_/Δf_casein_) × 100%. As can be seen from Figure 6, during the first 9 h, the changes in cleavage were similar and corresponded to removal on average of 27.8 % of the β-casein layer. By 18 h after plasmin addition, almost half of the β-casein layer was removed from the surface of the QCM transducer. This may be due to possible plasminogen activation in the sample.

**Figure 7:**
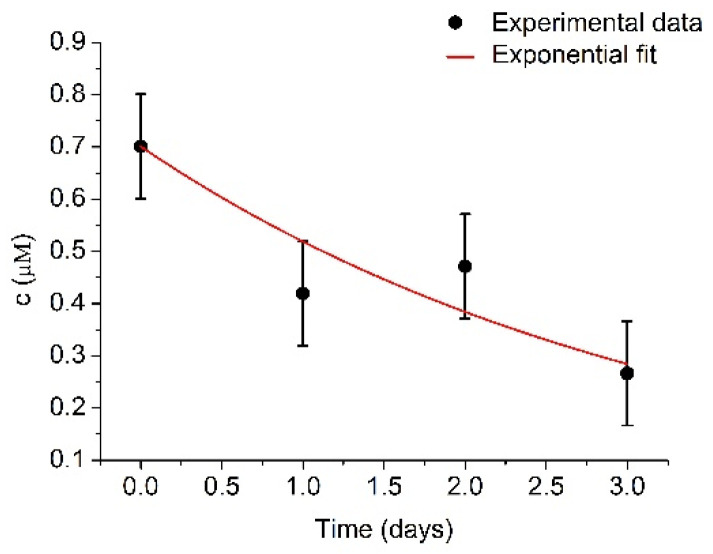
Changes of plasmin concentration, c, during several days measured by UV-Vis spectroscopy using spectrozyme-PL as a substrate. Initial plasmin concentration was c_max_ = 700.9 ± 99.8 nM and k = 0.30 ± 0.06 day^−1^. Results represent mean ± S.D. obtained from 4 independent experiments.

In order to provide further insights on the mechanisms of the decrease of the activity of plasmin over time, the changes in the concentration of plasmin determined spectroscopically using spectrozyme-PL as a substrate were determined. UV-Vis measurements require a larger quantity of plasmin in comparison with RQCM method. Despite this, four series of spectrozyme-PL cleavage by plasmin were performed. The measurements of plasmin concentrations were performed immediately, and then each subsequent day directly after plasmin activation. However, for better accuracy, a much higher concentration of plasminogen (1 μM) was used. Determined plasmin concentrations are presented at Figure 7.

The initial plasmin concentration was estimated to be 700.9 ± 99.8 nM and k = 0.30 ± 0.06 day^−1^. Plasmin concentration is relatively close to the concentration of the plasminogen (1 μM). The difference could be caused by prolonged storage. After fitting the experimental data by exponential curve, the concentration of plasmin decreased to half of its original value in t_1/2_ = 2.31 ± 0.46 days, which further confirms the expected plasmin half-life value. There was no recorded concentration increase in the first two days, as was in the case with the RQCM method, as the samples were older. Another possible reason could be more extensive plasmin autolysis at high plasmin concentrations [50]. This uncertainty presents certain limitations for use of the RQCM method, but it could be resolved by combining RQCM with optical or electrochemical methods.

## 4. Conclusions

It has been demonstrated for the first time that research quartz crystal microbalance (RQCM) is a very sensitive and promising method for studying the unusual characteristics of the inhibition of the proteases (trypsin and plasmin) by α_2_-antiplasmin as well as plasmin autolysis. We showed that addition of the protease: antiplasmin complexes at the surface of a β-casein layer immobilized at a QCM transducer resulted in smaller changes in the resonant frequency, allowing direct measurement of the degree of antiplasmin inhibition. The RQCM method can be used also to study plasmin autolysis, in long term experiments following addition of only plasmin (without inhibitor). In these experiments the changes in frequency increase which corresponds to a reduction in mass of the β-casein layer. This is due to the autolysis of the plasmin during its long-term storage (several days). The results provide evidence that RQCM is a promising tool for study of the mechanism of autolysis and protease inhibition. This method may also be useful for monitoring the properties of protein-containing solutions, including but not limited to dairy products.

## Figures and Tables

**Table 1:** The changes in resonant frequency, Δf, and surface mass density, Δm/A, caused by 5 nM trypsin and 5 nM plasmin applied at the surface of β-casein layers in the presence of various concentrations of α_2_-antiplasmin, and the degree of inhibition ε_i_ of protease activity calculated from kinetics of the frequency changes as ε_i_ = 100 × (ν_0_ − ν_i_)/ν_0_, where ν_0_ and ν_i_ are coefficients of the rate of hydrolysis of β-casein by plasmin without and in the presence of certain antiplasmin concentrations (see Supplementary Material for determination of the ν = df/dt value). Results are mean ± SD obtained from 3 independent experiments in each series.

α_2_-Antiplasmin (nM)	Trypsin			Plasmin		
	Δf (Hz)	Δm/A (μg/cm^2^)	ε_i_ (%)	Δf (Hz)	Δm/A (μg/cm^2^)	ε_i_ (%)
0	28.97 ± 0.31	−0.513 ± 0.005	0	19.8 ± 1.01	−0.350 ± 0.018	0
1.25	23.94 ± 0.36	−0.424 ± 0.006	15.29	16.26 ± 1.37	−0.288 ± 0.024	28.17
2.5	15.46 ± 0.8	−0.274 ± 0.014	49.48	14.4 ± 0.13	−0.255 ± 0.02	49.73
3.75	12.59 ± 0.47	−0.223 ± 0.008	80.06	3.9 ± 0.65	−0.069 ± 0.011	75.84
5.0	−3.98 ± 0.6	0.07 ± 0.01	100	−3.11 ± 0.81	0.055 ± 0.014	100

## Supplementary Materials

The following are available online at https://www.mdpi.com/article/10.3390/mi13010055/s1. Figure S1. The plot of the resonant frequency, f, vs. time following addition of plasmin (5 nM) at the surface of the piezoelectric transducer covered by β-casein. Figure S2. The plot of plasmin concentration vs. time of application after the activation (leaving out the first day of concentration increase.
