# Supplementary material for: New Insights on Plasmin Long Term Stability and the Mechanism of Its Activity Inhibition Analyzed by Quartz Crystal Microbalance
Source: Micromachines (Basel). 2021 Dec 29;13(1):55. doi: 10.3390/mi13010055 (PMC8777901; doi:10.3390/mi13010055)
Supplement: Supplementary file 1 [file micromachines-13-00055-s001.zip › micromachines-1508663-supplementary.pdf]

New Insights on Plasmin Long Term Stability and the Mechanism of its Activity Inhibition Analyzed by Quartz Crystal Microbalance

Marek Tatarko, Ilia N. Ivanov, Tibor Hianik

1. Determination of the Degree of Protease Inhibition

Proteases plasmin and trypsin were diluted in 10 mM phosphate buffered saline (PBS), pH 7.4 on required concentration. α_2-antiplasmin was added from stock solution of 1.43 μM concentration into the protease sample. Antiplasmin final concentration in each protease sample was in the range from 0 to 5 nM. The solution was stirred and incubated for 20 min at room temperature before being added at the sensor surface modified by β-casein layer with a flow rate of 50 μL/min.

The degree of inhibition, ε_i (in %), has been calculated from the kinetics of the frequency changes using equation (1):

$$\varepsilon_i = 100x(\nu_0 - \nu_i)/\nu_0, \qquad (1)$$

where ν_0 and ν_i are coefficient of the rate of hydrolysis of β-casein by plasmin without and at present of certain concentration of antiplasmin. This coefficient can be determined from the kinetics of the changes of the resonant frequency, f, using the equation:

$$f= f_{max}t^n/ (\kappa^n+t^n), \qquad (2)$$

where f_{max} is final, steady-state resonant frequency value after addition of plasmin or plasmin-inhibitor complex, while constant κ and Hill coefficient n are determined by fitting the plot of the resonant frequency vs. time as it is shown on Figure S1. In order to determine the rate of hydrolysis, ν_i, fitted curve

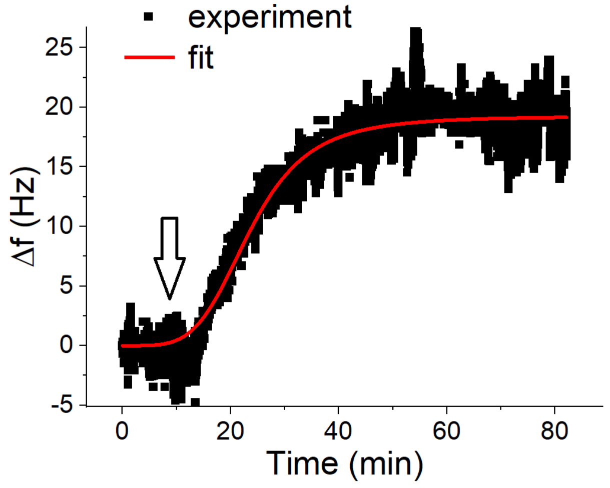

Figure S1. The plot of the resonant frequency, f, vs. time following addition of plasmin (5 nM) at the surface of the piezoelectric transducer covered by β-casein. The red curve is fit according to Eq. (2) using OriginPro version 7.5 (OriginLab Corporation, MA, USA). The fitting parameters were as follow: f_{max} = 19.24 ± 0.01 Hz, κ = 23.57± 0.01 min, n = 4.33 ± 0.01. The moment of plasmin addition is shown by arrow.

was derivate by time using OriginPro. Increasing linear part of the derived curve was isolated and fitted by linear fit by Origin Pro to find value of the linear curve slope - value of the coefficient ν_i. In the case of curve presented in Figure S1, ν_i = 0,088 ± 1.714×10^{-4} Hz/min^2.

2. Determination of the Plasmin Shelf Half-life

After plasminogen activation, undiluted plasmin (≤ 1 μM) was stored at 4 °C in the fridge for 2 weeks. This stock solution was used for preparation 5 nM plasmin in PBS 30 min. before experiment. Plasmin has been added at the surface of the β-casein layer immobilized at QCM transducer. The kinetics of the frequency was evaluated and total change of frequency value after 30 min. application was recorded. The first graph was constructed by plotting total frequency change for each day of the measurement and for both series.

Concentration values for the second graph were estimated by setting the initial concentration value to 5 nM and changing other values to have concentration proportional to the first one. This exchange was also supported by spectroscopic concentration determination. The plot of plasmin concentration vs. time of application after the activation is shown on Figure S2.

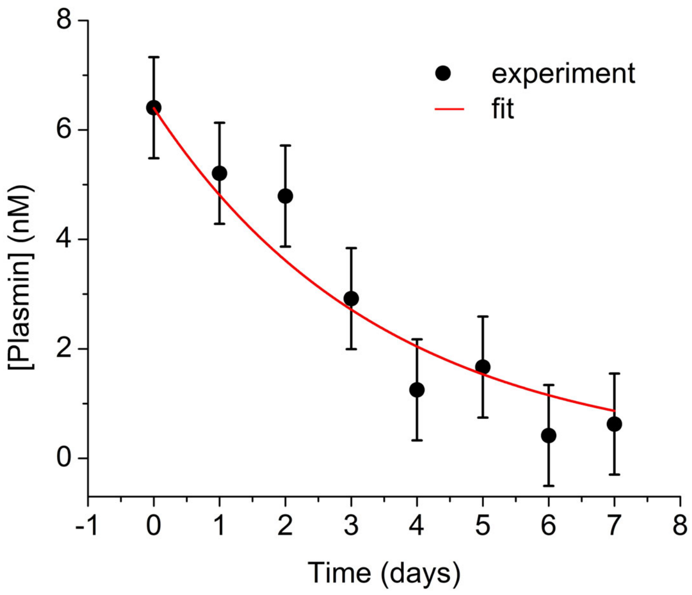

Figure S2. The plot of plasmin concentration vs. time of application after the activation (leaving out the first day of concentration increase). The red curve is fit according to Eq. (3) using OriginPro version 7.5 (OriginLab Corporation, Northampton, MA, USA). The fitting parameters were as follow: c_{max}= 6.41 ± 0.92 nM and k = 0.29 ± 0.03 day^{-1}.

Transformed concentration values were fitted by the exponential equation:

$$c = c_{max}\, e^{(-kt)}, \qquad (3)$$

where c is applied plasmin concentration, c_{max} is the initial maximal concentration and k is the reaction rate (see red curve at Figure S2).

The first day, when concentration increased, has been omitted from the fit. Exponential fitting allowed us to determine value of the exponential coefficient – reaction rate of decrease of plasmin k = 0.29 ± 0.03 day^{-1}. Plasmin shelf half-life was determined by using k and standard half-life formula (4) for both series:

$$t_{1/2} = \ln 2 / k \qquad (4)$$

Calculated value $t_{1/2}$ = 2.48 ± 0.28 days was compared to the plasmin shelf half-life values from literature and from the spectroscopic measurements (see the main text).
